# Suppression of Psychological Needs Among Beginning Teachers: A Self-Determination Theory Perspective on the Induction Process in Bedouin Schools

**DOI:** 10.3389/fpsyg.2021.621984

**Published:** 2021-03-15

**Authors:** Haya Kaplan

**Affiliations:** Kaye Academic College of Education, Be'er-Sheva, Israel

**Keywords:** autonomy support, autonomy suppression, beginning teachers, emotional-motivational experiences, induction period, self-determination theory, Bedouin society

## Abstract

The study focuses on the emotional-motivational experiences of Bedouin-Arab beginning teachers during the induction period, from the perspective of Self-Determination Theory. A phenomenological study was employed. Seventy-four teachers participated, 62 of whom completed open questionnaires, while semi-structured interviews were conducted with 12 other participants. The findings indicate that the beginning teachers reported experiences of coercion, exploitation, and gender-based discrimination (autonomy suppression). They also experienced a judgmental attitude, lack of assistance, and difficulties with students (competence suppression), and their sense of relatedness to the school is impaired due to cultural factors (relatedness suppression). As a result, they expressed controlled motivation, a sense of burnout, stress, impaired well-being and disengagement in school. They also suppressed their students' autonomy. At the same time, the findings also show that when the teachers experience a sense of need satisfaction, they integrate well into the school. These findings indicate the necessity for establishing a need-supportive school environment for beginning teachers during their induction period.

## Introduction

“I sat in the corner of the room and tried to make myself invisible…The principal continued berating me in front of everyone; all the teachers in the school were sitting there, and he's shouting and reprimanding me…” This account, by a teacher in her internship year at the school, expresses a profound experience of suppression.

As an educator and researcher who has been active in Bedouin schools for many years, I am not a stranger to the voices of beginning teachers/interns describing their experiences during their induction period. The study seeks to give these voices a platform in order to bring about change.

The conceptual framework, employed to gain an understanding of the experiences of Bedouin beginning teachers/interns, is ground in self-determination theory (SDT) (Deci and Ryan, 2000; Ryan and Deci, 2017), and more specifically, basic psychological need theory (BPNT), one of the six mini-theories within SDT (Vansteenkiste et al., 2020). SDT highlights people's psychological needs as inherent motivational assets. According to SDT, people have three basic psychological needs: (1) for relatedness; (2) competence; and (3) autonomy (Deci and Ryan, 2000). Education research shows that needs-supportive settings and perceived needs satisfaction contribute to positive outcomes among both students (e.g., Jang et al., 2009; Assor et al., 2018; Kaplan, 2018) and teachers (e.g., Roth et al., 2007; Roth, 2014; Cheon et al., 2020).

The study focuses on teacher motivation. Most SDT-based investigations on teacher motivation have been conducted among in-service teachers (e.g., Aelterman et al., 2016) and pre-service teachers (e.g., Evelein et al., 2008; Perlman, 2015; Kaplan and Madjar, 2017). Despite an extensive body of research showing the complexity of the induction process (De Neve and Devos, 2017), there are comparatively few SDT-based studies on beginning teachers' motivation (Fernet et al., 2016; Kaplan et al., 2016).

In addition, previous studies have demonstrated the applicability of SDT in diverse cultures (e.g., Jang et al., 2009), but only a few have scrutinized Bedouin culture (Kaplan et al., 2014; Kaplan and Madjar, 2017; Kaplan, 2018; Kaplan and Assor, 2019), specifically among Bedouin beginning teachers/interns. The Bedouin world is a collectivist-patriarchal society with unique features that are reflected in relationships in the schools (Arar and Oplatka, 2013; Sharabi, 2014); hence the importance of delving into the experiences of beginning teachers/interns in Bedouin schools.

Within the domain of teacher motivation, the vast majority of SDT-based studies have been quantitative (Han and Yin, 2016). It is only recently that a call has been issued to complete qualitative SDT-based studies as well (Chirkov and Anderson, 2018).

The following questions guide the present study: What characterizes the emotional-motivational experiences of Bedouin beginning teachers/interns, and what conditions at the school can either promote or hinder experiences of needs satisfaction in their culture?

I will begin by describing the world of beginning teachers/interns. I will then review the field of teacher motivation, and the environmental features that support or hinder these psychological needs. Then the study's cultural context will be presented.

### The World of Beginning Teachers/Interns: From Dreams to Survival

Young teachers enter education imbued with a sense of mission, and a desire to bring about change in society and their chosen field. They discover very quickly that the “real” world of their school and teaching differs considerably from the one depicted in their educational worldview and expectations (Arar and Masry-Harzallah, 2016).

The professional literature indicates the obstacles faced by beginning teachers/interns (Pritzker and Chen, 2010). For example, Pritzker and Chen (2010) reported on heavy teaching workloads, difficulties in relationships with management and fellow teachers at school, and the “reality shock” that accompanies beginning teachers/interns' encounter with their schools. Arab beginning teachers also experience challenges during their induction period, in addition to which, they have to contend with the complex reality of the Arab education system, and the centralized educational climate in many schools (Watad Khoury, 2013).

Coping problems at this stage are likely to lead to teachers dropping out of the education system, a troubling phenomenon in many countries around the world, including Israel (Sperling, 2015). Dropout rates in Israel's Arab schools have risen in recent years, although they are still low compared to those of Jewish schools, since Arab teachers do not have alternative employment options. Consequently, it is vital to examine the quality of teachers' experiences and motivation in the Arab schools.

### SDT and Teacher Motivation

According to SDT (Deci and Ryan, 2000; Ryan and Deci, 2017), people have three basic, universal psychological needs, mentioned earlier: (1) relatedness; (2) competence; and (3) autonomy. The need for relatedness refers to teachers' experience of having close, safe, and satisfying relationships with others at the school and in the community (the principal, senior teachers, students, parents, and others). The need for competence is the teacher's experience of effectance and mastery in his/her educational practice; viewing himself/herself as capable of realizing intentions, plans, and objectives. The need for autonomy concerns the experience of self-determination, authentic self-expression, meaning, and freedom of choice.

The key to optimal functioning in teaching—which entails autonomous motivation, quality engagement, and emotional and social adjustment—is an experience of needs satisfaction (Roth, 2014). The social context plays an important role in supporting such a need (Deci and Ryan, 2000). Based on previous studies (e.g., Assor et al., 2002; Reeve, 2006; Kaplan and Madjar, 2017), relatedness support includes a warm attitude, displaying care and concern, and expressing interest in beginning teachers/interns' activities. Competence support includes behaviors such as providing structure (i.e., a “roadmap” of the school, its requirements, and procedures), clarifying expectations, setting ideal challenges, and offering informative feedback. Autonomy support encompasses conduct such as acknowledging beginning teachers/interns' unique opinions, enabling them to have a choice, encouraging initiatives, involvement in making decisions, and refraining from coercion (e.g., using controlling language, placing demands without an explanation, and so forth).

SDT distinguishes between autonomous and controlled motivation (Deci and Ryan, 2000). Teachers with autonomous motivation experience a sense of profound satisfaction from teaching (intrinsic motivation). They identify with the profession and its practices, and see the connection between engaging in teaching and their personal values, goals, and abilities (identified regulation). Teaching is part of their identity, and they experience a sense of will, self-actualization and meaning (integrative regulation). Teachers with controlled motivation act out of external regulation (e.g., fear of the principal's reactions) or out of introjected regulation (i.e., internal pressure, a sense of coercion, and negative emotions).

In the past decade, the domain of teacher motivation has drawn scholarly attention, especially with regard to the motivation of in-service teachers (Roth, 2014). Supporting teachers' needs, and their experiences of needs satisfaction, are associated with a variety of positive outcomes e.g., autonomous motivation in teaching (Eyal and Roth, 2011; Kaplan and Madjar, 2017), self-actualization (Kaplan and Madjar, 2017), and more. Teachers' autonomous motivation is tied to outcomes such as greater engagement in work (in de Wal et al., 2014), a sense of self-actualization, and a reduced sense of burnout (Roth et al., 2007), along with supporting students' autonomy (Roth et al., 2007).

An experience of needs frustration predicts controlled motivation and a range of negative outcomes among teachers (Vansteenkiste and Ryan, 2013). Needs frustration can manifest in the behaviors of numerous figures, demands, procedures, and routines at school, which do not enable teachers to act in a way that fulfills their needs (Pelletier et al., 2002; Reeve, 2009). The principal's support is perceived as a major component in promoting teachers' need-satisfaction (Klassen et al., 2012).

Studies have indicated the significance of a needs-supportive environment among pre-service teachers as well (e.g., Evelein et al., 2008; Perlman, 2015; Kaplan and Madjar, 2017). A few investigations have also been conducted among beginning teachers (e.g., Fernet et al., 2016; Kaplan et al., 2016).

The reviewed studies point to the need to continue exploring the motivation of beginning teachers. In the present study, I delve into the experiences of needs satisfaction and frustration among Bedouin-Arab beginning teachers/interns.

### The Bedouin Society and the Bedouin Education System

The Bedouin society is a hierarchical-collectivist and patriarchal community (Sharabi, 2014). In collectivism, individuals are united in a membership group typified by norms and practices that accord preference and precedence to the group's interests and objectives over the individual's goals and needs. The communication style dictates acceptance of the group's aims; consequently, it is more authoritative. The social structure of the Bedouin society is unique and based on tribalism, which is characterized by loyalty to the membership group (i.e., family, tribe), strictly maintaining the value of honor, a rigid hierarchical arrangement, and a high level of obedience to male and parental authority figures (Alsayed, 2015).

In recent decades, substantial changes have occurred in the lives of Israel's Bedouins. Social and cultural changes resulted from urbanization—as Bedouins have gradually abandoned the nomad lifestyle and moved into permanent settlements—and from exposure to Western culture and its values. These changes include, for example, increasing rates of young Bedouins, especially women, seeking higher education; growing numbers of women in the job market (particularly in education); and modernization of the tribal life style.

However, despite these shifts, the Bedouins' way of life can still be defined as traditional and collectivist (Sharabi, 2014; Nasser-Abu Alhija and Israelashvili, 2021).

The schools reflect the society and its traits, and relationships with teachers resemble ties with authority figures in the family (Abu Asbah, 2006). Teaching methods exhibit the prevalent patriarchal-collectivist patterns in Bedouin culture (Abu Asbah, 2006; Iliyan, 2013). School principals in the Bedouin education system function in a reality rife with contradictions. On the one hand, they operate in accordance with the Ministry of Education's guiding principles and procedures; on the other, family and tribal pressures driven by cultural considerations are exerted on them (Arar and Masry-Harzallah, 2016), leading them to act in a manner that diverges from the norms of the Israeli education system. A study conducted by Arar and Oplatka (2013) indicates that Arab school principals adopt a centralized, controlling leadership style.

With reference to the cultural aspects of Bedouin schools, and further to claims made by researchers holding a cultural relativism approach (e.g., Iyengar and DeVoe, 2003), and contrary to the assertions of SDT (Deci and Ryan, 2000), one could argue that in collectivist, patriarchal societies (e.g., the Bedouin community), the need for autonomy would be of lesser importance to beginning teachers, and the effects of autonomy suppression would be less acute (e.g., Iyengar and DeVoe, 2003; Liu and Flick, 2019). As stated earlier, Bedouin beginning teachers/interns experience difficulties during their induction period (Watad Khoury, 2013). Yet to date, these challenges have not been examined from the angle of BPNT (Vansteenkiste et al., 2020). From an SDT perspective, it is interesting to look at how cultural characteristics manifest in the nature of the hurdles these teachers face.

### The Present Study

The research questions are: What characterizes the emotional-motivational experiences of beginning teachers/interns in Bedouin schools from the standpoint of basic psychological needs satisfaction or frustration, and what conditions at school can promote an experience of needs satisfaction?

In the realm of teacher motivation, most SDT-based studies have been conducted within a positivist paradigm (Han and Yin, 2016; Chirkov and Anderson, 2018). Han and Yin (2016) and Chirkov and Anderson (2018) maintained that studies anchored in the positivist paradigm do not facilitate a deep understanding of the complexity of motivational processes and mechanisms. Chirkov and Anderson (2018) asserted that, “Psychologists need to study motivation where it is actually happening—in the embodied human beings that are embedded into historical and sociocultural contexts…” (p. 730).

Following this call, the study employed a qualitative paradigm within the phenomenological genre (Creswell and Poth, 2017), and sought to investigate the experiences of Bedouin beginning teachers/interns during their induction period (the phenomenon), and the meaning they assign to these experiences.

## Methodology

### The Participants and Context of the Study

Seventy-four teachers participated (a purposeful sample), of whom 34 were interns, and 40 were beginning teachers; 61 were female, and 13 were male. Sixty-two participants completed open questionnaires and 12 others were interviewed. All of the participants belong to the Bedouin-Arab population. At the time of the study, 42 of the participants were working in elementary schools, 20 in junior high and high schools, and 12 in special education institutions.

Five of the interviewees were interns, and seven were beginning teachers; six were working in elementary schools, and six in high schools (12 different schools). The 62 participants who completed the questionnaire worked in 58 different schools.

The study was conducted as part of the activities of an academic induction unit at a college of education in which SDT serves as an organizing conceptual framework. SDT principles are implemented in workshops for teacher interns, beginning teachers, and for veteran teachers training to become teacher-mentors. The study emerged from the need to gain closer familiarity with the inner world of the teachers we work with in the college, in order to focus our work on their needs.

### Research Tools

To increase the study's trustworthiness, two data collection methods were employed: (1) an open questionnaire and (2) semi-structured interviews (Shkedi, 2003). The open questionnaire enabled a latitudinal view of a relatively large number of participants. However, an open questionnaire can hinder an in-depth writing of experiences. In addition, open questionnaires do not enable the researcher to ask the respondents to elaborate. Consequently, in the second stage, semi-structured interviews were conducted in a safe setting, which allowed the interviewees to address the questions in greater depth, and the interviewers to ask the participants for elaborations.

“The use of multiple methods, or triangulation, reflects an attempt to secure an in-depth understanding of the phenomenon in question” (Denzin and Lincoln, 2008, p. 7). The combination of multiple approaches adds to the study's richness; this helps to enhance its trustworthiness and serves as an alternative to validation (Shkedi, 2003; Tsabar-Ben-Yehoshua, 2005; Denzin and Lincoln, 2008). The research tools are described below.

#### Open Questionnaire

The questionnaire centered on the beginning teachers/interns' induction experiences. The participants were given the following instruction: “Speak about a significant experience you had this year at the school as an intern/beginning teacher that represents the absorption process at the school, an experience you feel will be engraved in your memory (positive or negative).” They were asked to elaborate: “Describe the experience. What made it significant for you? What permitted this experience to occur? Who was involved and how? How did you react? What helped you cope?”

#### Semi-structured Interview

In the first part of this interview, the participants were asked to speak about a significant experience associated with their induction period. This part was identical to the open questionnaire described above. The interview included an additional section that allowed them to go into detail about their internship year or the year following it. This part began with an open question: “Tell me your story as an intern/beginning teacher.” Then the interviewees were asked to provide particulars on topics such as their experience of teaching in the present, their relationships with different figures, their goals in teaching, successes or challenges, and their aims for the future.

### Procedure

The data collection procedure consisted of two stages. In the first, the open questionnaire was administered in the college workshops. The participants were told that completing the questionnaire was optional and anonymous.

The workshops took place at the college during the academic year. Interns' workshops had 60 h of weekly sessions, whereas beginning teachers' workshops had 40 h of biweekly sessions. Questionnaires were handed out on the seventh session, to allow attendants more teaching experience prior to participating in the study (they started working in September and the workshops began at the end of October). The questionnaires were administered in four randomly selected groups, attended by 80 teachers. About one or two teachers in each group did not participate either because they were absent from the particular session or chose not to take part. The participants completed the questionnaire in ~30 min.

In the second stage, after the administration of the questionnaires had been completed, 12 interviews were conducted with beginning teachers/interns who had not completed the open questionnaire. The interviewees came from two additional groups, one of interns and one of beginning teachers. In each group, about ten teachers agreed to participate. The 12 interviewees were randomly selected, maintaining a balance between interns and beginning teachers.

Beforehand, the interviewer explained the topic of the interview, asked for permission to record it, and assured the interviewee that none of the issues that came up would be passed on to anyone at his/her college or school. Each interview lasted ~45 min.

Importantly, although the workshops followed SDT principles (through need-supportive guidance), the conceptualization of the theory's principles was done after the interviews and questionnaires had been completed. Thus, at the time of the data collection, the participants did not know about Self-Determination Theory or the concept of basic psychological needs, and the content of the workshops could not have affected the results.

### Data Analysis

A thematic analysis was performed for both the questionnaires and the interviews. To enhance the trustworthiness of the study, the analysis process involved two independent raters: the author of this paper, who has read and analyzed the transcripts of all questionnaires and interviews; and a colleague who assisted in the analysis, which focused on checking the interrater agreement. Both scholars are familiar with Self-Determination Theory and both are highly experienced in content analysis and qualitative research methods.

The analysis included three stages (Shkedi, 2003; Creswell and Poth, 2017). In the first stage, we conducted a holistic reading of each interview and questionnaire. In the second stage, the main themes were derived (through an analysis of each of the open questionnaires and interviews). The analysis was based on the research questions and the theoretical framework (SDT concepts and the nature of Bedouin culture). Consequently, a criteria-based content analysis was employed (Shkedi, 2014). A directed approach to content analysis is widely used to probe qualitative content (Hsieh and Shannon, 2005). Hsieh and Shannon (2005) contended that an existing theory about a phenomenon can guide the coding scheme or the relationships between codes. In accordance with this approach, through deductive analysis, we classified participants' responses into the various themes. When a particular response expressed two separate themes, it was classified into each of them.

**Figure 1:**
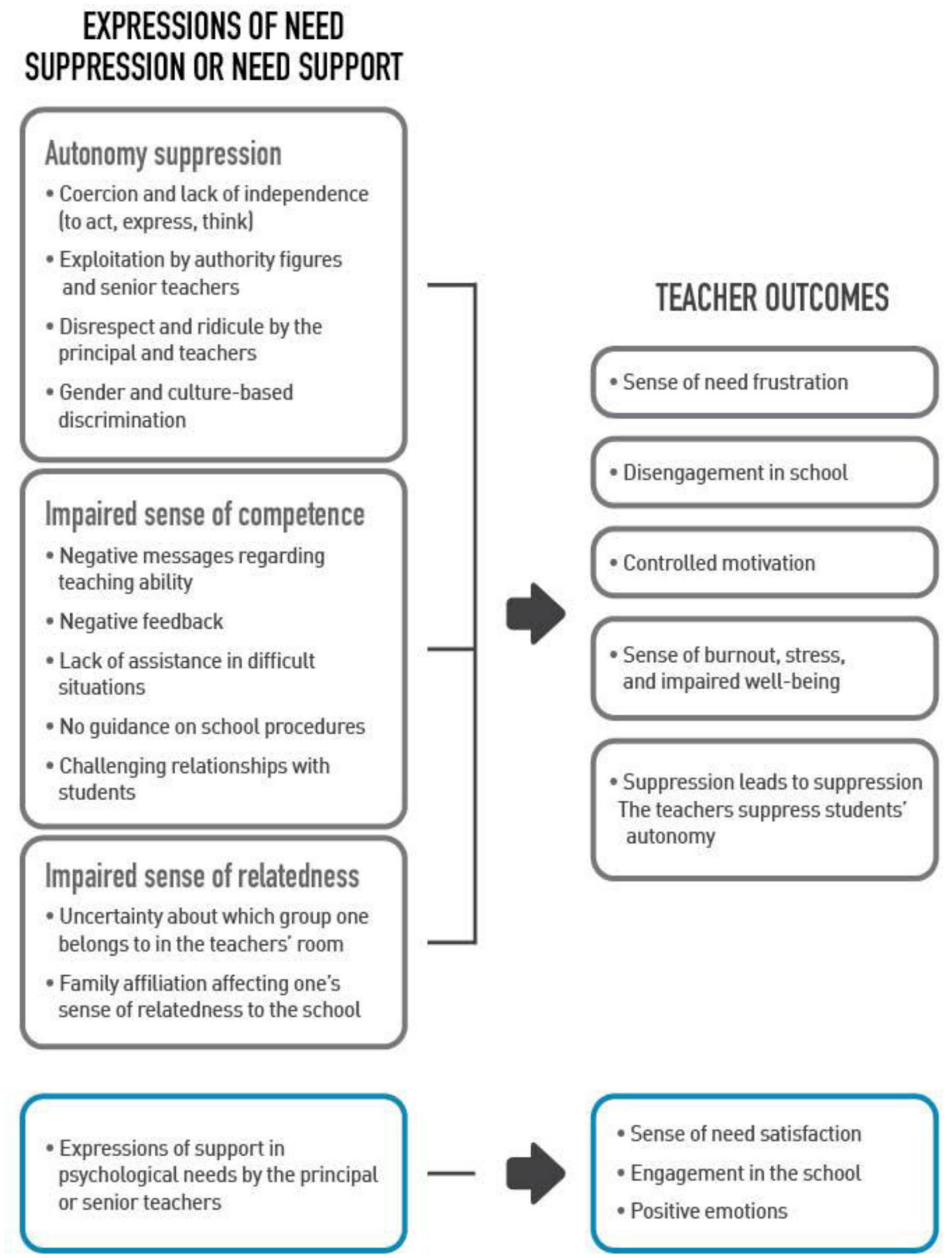
The experiences of beginning teachers in Bedouin schools: Finding mapped in a category tree.

In the third stage, the analysis focused on reorganizing the themes, finding sub-themes, identifying connections, and creating the final set of themes (Shkedi, 2003; Creswell and Poth, 2017). This process was concluded by drawing a category tree that demonstrated the central themes and the connections between them (see Figure 1).

This method facilitates an examination of SDT's applicability to beginning teachers/interns in the Bedouin society and reveals SDT's unique cultural manifestation from the participants' subjective-phenomenological outlook.

During the analysis, the author and her colleague conducted an initial evaluation of randomly selected 20% of the transcripts to assess interrater agreement. Unclear themes during initial coding by the author and her colleague as well as cases where there was disagreement were discussed to reach consensus. Interrater agreement was measured through percent agreement method (McHugh, 2012). The author and her colleague reached a consensus on 84% of these items at this preliminary phase. This high consensus rate can be ascribed to the fact that both scholars are knowledgeable in Self-Determination Theory.

## Findings

Scrutiny of the overall picture of positive and negative experiences described in the interviews and in the responses to the questionnaire shows that for 54 participants (73%), their dominant experience is negative. Negative experiences emerged in nine interviews (out of 12), and in 45 responses to the questionnaire (out of 62). The other 20 participants (27%) said they have received support and assistance from the principal or fellow teachers since they started working at their respective sites.

The analysis yielded the following themes: **Theme 1** relates to suppressing the need for autonomy. **Theme 2** involves suppressing the need for competence. **Theme 3** is associated with suppressing the need for relatedness^1^. **Theme 4** entails the implications of psychological needs frustration. **Theme 5** speaks to the bright side of the story—needs support received from principals and teachers. In the following section, the themes are described in detail (all of the names mentioned in the Findings section are fictitious).

Please note that while the quotes that appear below are classified into themes according to the three basic psychological needs, some quotes may fit more than one need, in addition to the one at the center of the theme.

### Theme 1: Suppressing the Need for Autonomy During Induction

#### The Experience of Coercion and a Lack of Freedom (to Act, Express, Think)

Nineteen participants (26%) reported that they have experienced autonomy suppression from the principal and teachers at their school. Examples include imposing opinions, dictating assignments and programs, not providing choice, non-involvement in making decisions, not listening, restricting the free expression of ideas and opinions, and suppressing creativity. The participants used expressions, such as “prison” or “there's no room for me in the program” or “my principal is a control freak.” The following quotes convey their sentiments:

**Maram (a beginning teacher during her interview):** My principal is a control freak who's constantly trying to impose his opinion on his teachers, and he's always telling us to do what he says because he's more experienced than we are. He's not considerate of us as human beings who might fall ill or get tired. He wants us to be like clocks and expects us to listen to him when he doesn't listen to any of us…This frustrates me. This isn't a school, it's a prison.

Other teachers referred to non-involvement in making decisions, which causes them to feel alienated and impairs their sense of relatedness to their school. In her response to the questionnaire, Najat, a beginning teacher, remarked:

Contact with the principal happens via the noticeboard. Everything is imposed from above as a fait accompli. The teachers, especially the beginning teachers, aren't involved in making decisions. We work and work at the school, so I don't feel any affection for the school or relatedness to it.

#### Experiences of Exploitation by Authority Figures and Veteran Teachers

Eighteen participants (24%) mentioned cases of exploitation by their principal, veteran teachers, and various authority figures. They described feeling silenced and invisible. The exploitation exhibits in abuse of authority and worsening employment conditions (e.g., scattered teaching hours throughout the week and placing teachers in classes with difficult students). The exploitation stems from a sense of unfamiliarity with the school's rules and procedures. The authority figures abuse their power and exploit the beginning teachers/interns' unfamiliarity with procedures. This causes frustration and feelings of humiliation. For example, Badour, a beginning teacher, described the exploitation of beginning teachers/interns by a member of the school's leadership in an interview:

The vice-principal is pathetic [not assertive]. He doesn't do anything, the veteran teachers take advantage of him and the school, and aren't prepared to do anything, and he takes advantage of us. And that's not good. Why doesn't he ask the veteran teachers to substitute? Why only us? I wonder about this treatment, I mean, we're all teachers! We do the individual teaching hours, and they don't? There's exploitation here. I'm really frustrated.

Two interns noted that the exploitation they experience stems from social or cultural reasons. The authority figure exploits information he/she possesses regarding the teacher's clan affiliation.

**Samah:** I'm a beginning teacher and I have to teach in my village. I have a half-time position, but I come to school every day because my hours are scattered across the whole week. The principal knows I can't teach anywhere else! My husband won't allow it. He takes advantage of my family situation. He knows I don't have other options (Intern, questionnaire)

There is also exploitation by teachers who perform various functions (e.g., they are the scheduling or subject coordinator), which emerges through unequal treatment and discrimination against beginning teachers/interns. In her interview, Amira, a beginning teacher, commented:

You feel like a robot, nothing! You can't do anything, the school doesn't trust you and even exploits you…I know that an individual teaching hour [involves] teaching one child, but all of a sudden, you're told to go and teach an entire class. If I'm absent, everybody looks at me oddly and tells me I'm not allowed to be absent, even though according to logic I'm allowed to be absent… It's exploitation and I'm fed up with them, I haven't got the strength for it anymore…

#### Displays of Disrespect and Ridicule Toward Beginning Teachers/Interns

Nine participants (12%) reported being subjected to ridicule, disrespect, intolerance, a lack of empathy, and insensitivity from the principal and veteran teachers. They feel offended, and that their self-esteem has been weakened. In addition to impairing the teachers' sense of autonomy, these experiences also undermined their senses of competence and relatedness to the school. In her interview, Amira (mentioned earlier) described her first encounter with the entire teaching staff and how she was introduced to them:

He [the principal] looked at me disapprovingly and so did the teachers. Even though I told them I was new and didn't know the schedule or the environment, none of them bothered to invite me to sit down. I sat on the edge of the chair, half sitting! I wanted to run away; I was so embarrassed and offended. At the end of the meeting, the principal said: “This is the new teacher, try to make correct use of her,” and I didn't understand what he meant! And the Hebrew coordinator said: “Ah, you're the new one—the new broom!” I felt completely out of it.

#### Gender and Culture-Based Discrimination

Ten participants (13%) spoke of a discriminatory, unjust, and harsh attitude by the principal or other authority figures based on them being young women. They mentioned feeling alienated, and perceive themselves as a minority at their school. Some participants believe they are treated differently due to their cultural affiliation (e.g., being from a different tribe or region [locality]). These suspicions cripple their sense of relatedness.

In her interview, Sireen, a beginning teacher, wondered how she could have a sense of relatedness when there are so many sanctions and restrictions at her school. She said that the principal has explicitly stated his preference for male teachers:

How can I be connected to the school? The principal is always shouting and threatening. Once he even said he doesn't prefer female teachers because they're [women]…he was blunt with me at my interviews, and asked me if I planned to get pregnant. That's the height of audacity and a violation of privacy.

In her interview, Samer said she feels like a stranger at her school. The principal does not treat her fairly because she is female:

The principal's attitude is unfair and he distinguishes between the teachers. Being a woman and from a different locality positions me as someone who has no worth. I don't feel confident, and I don't understand the school rules. I'm a minority [woman] within a minority [beginning teachers]. I'd rather be on the side and not belong to the school.

### Theme 2: Impairing the Need for Competence

#### An Absence of Guidance on School Procedures (e.g., Rights, Forms, Role Definitions, Etc.)

Twenty-three participants (31%) commented that negative experiences have made them feel they are not yet ready for the world of school. They have difficulty understanding the procedures, the messages they receive are confusing, they feel they do not know how to conduct themselves at the school, and they experience ambiguity in the definition of their role, as expressed by Maram in her response to the questionnaire.

The teachers made me feel I'm not mature enough or ready to teach at the school. About everything they'd say: What, you don't know? Even the simplest things, like the mapping assessment. I wish I'd taken a part-time job, or that I wasn't a homeroom teacher.

#### Negative Messages Regarding Teaching Ability, Negative Feedback, a Judgmental Attitude, and Failure to Provide Assistance in Difficult Situations

Twelve participants (16%) reported not receiving competence support from the principal or veteran teachers. The need for competence is obstructed by means of negative, judgmental messages regarding the beginning teachers/interns' teaching or classroom management abilities, belittling them due to their young age, negative feedback following classroom observations, and not responding to their need for help. Competence suppression by the principal was prominent in the interview with Amira:

The principal came into my classroom. I was teaching and connecting with the students. After a few minutes, he left, and then the vice principal came and told me the principal wanted to talk to me. I went to see him after the lesson, and he told me he didn't know how I completed teachers' college, and that I was going in one direction and the students in another. That killed me; I felt I was choking, detached. I'll never like that principal… He wants me to teach the way he's been teaching for the past 40 years.

#### Challenges in Relationships With Students: Discipline Issues and Differences Between Students

Ten participants (14%) mentioned the student population and issues associated with them (e.g., discipline problems, differences between students, overcrowded classrooms, etc.) as hampering their sense of competence. Not receiving assistance from relevant figures at the school intensifies beginning teachers/interns' sense of helplessness, as demonstrated in the following quote from an interview with Asmaa, a beginning teacher:

The students here are really wild. They take advantage of me being new at the school, and they look bigger than me. It's hard for me to share my feelings with the principal. I don't want him to start thinking I'm not responsible or don't measure up to expectations.

### Theme 3: Suppressing the Need for Relatedness

#### The Beginning Teacher/Intern—Between Different Groups in the Staff Room

Eight participants (11%) referred to relationships in the staff room and “cliques” at the school. They find themselves in situations of conflict between different groups of teachers that belong to different families or tribes. Veteran teachers expect them to identify with a particular group, which engenders feelings of stress and embarrassment in them. They avoid connecting with other teachers, and their confidence is diminished. In her response to the questionnaire, Haneen, an intern asked: “Who should I belong to?”

It was already during the preparation day. I could see something was off, so I asked one of the veteran teachers I happen to know, and then I realized there were groups and cliques, even in the Arabic staff that I belong to, and it really made me nervous. Who should I belong to? And how will I maintain good relations with everyone?

#### The Effects of Family Affiliation on One's Sense of Relatedness to the School

Seven participants (9%) noted that their family and tribal affiliation affects their sense of relatedness to their school. In collectivist Bedouin culture, the loyalty of the membership group is first and foremost to one's tribe. In many schools, the body of students and teachers reflects the composition of the population in the school's locality, which often comprises several tribes and families. The findings show that perceived frustration surrounding the need for relatedness increases due to teachers' family affiliation.

Beginning teachers/interns who teach at a school in which they are a family minority have an impaired sense of relatedness, while those who teach at a school that “belongs” to their family experience a sense of relatedness, and are optimally absorbed into the school. The impact of family affiliation on one's sense of relatedness to his/her school is reflected in the following quotes from responses to the questionnaires by two beginning teachers.

**Jalal**: I'm not prepared to contribute to the school, and I don't feel I belong to it. I know how I'll be treated at the school because of the tension between my tribe and the principal's tribe around the [local] elections. To me it's just a workplace and I don't need to make friends. I prefer my tribe and it takes precedence over the school.

**Ameen:** I come from a small family, and I wanted to teach at the school. In our very first meeting I could already see the disrespect and disdain in the principal's eyes, like he doesn't have a positive view of me teaching at the school, as if the school belongs only to his family.

### Theme 4: The Implications of Suppressing and Frustrating Psychological Needs

#### The Implications of Autonomy Suppression for Beginning Teachers/Interns' Motivation

Negative experiences of coercion, exploitation, and belittlement have harmed beginning teachers/interns' motivation and their relationships with various figures at their schools (management, students, parents). They are reluctant to participate in activities and contribute to their schools. The negative emotions they express, and the overt statements regarding their unwillingness to be engaged in school life, allude to controlled motivation, and even amotivation. This theme manifested in the comments of 12 participants (16%).

**Adham (an intern, in his response to the questionnaire):** I prefer not to get involved in anything. If the veteran teachers don't accept me because I'm new and take advantage of me—for instance, as a substitute teacher or teaching weak classes—I'm not prepared to participate in the team-building day. It's wrong!

**Asmaa (a beginning teacher, in her response to the questionnaire):** I felt—and still feel—the exploitation. I haven't got the strength to argue, and I'm fed up with the whole school.

These ordeals engender a sense of guilt. The participants sometimes blame the environment for their situation, as expressed by Naima, a beginning teacher, in her response to the questionnaire:

It's disrespectful and illogical, and it's really offensive, I don't know what the reason is. Maybe the lenient principal or the exploiting vice principal? Or perhaps I'm to blame for the exploitation because I let them take advantage of me? I should have behaved differently.

In her responses to the questionnaire, Salma (an intern) mentioned her impaired sense of relatedness to the school, and explicitly stated that the treatment she receive at school hinders her motivation and creativity.

**Salma:** …I just don't feel like doing anything—they ruined my motivation and creativity. There's no room to introduce something of my own.

#### Suppression Leads to Suppression: The Teacher Suppresses Students' Autonomy as a Response to His or Her Experience

Seven participants (10%) said the suppression and subsequent frustration they have gone through has led them to replicate these experiences both within and outside of school, and to treat students—and even family members—as they have been treated.

**Huda (a beginning teacher during her interview):** I started discriminating against the students too. It's like it's transferred from the principal to me and from me to the students. I come into the classroom really irritated. I'm not prepared to hear anything from the students. I dictate, and I'll dictate everything. The problem isn't only at school, but at home too.

In contrast, some teachers noted that the support they receive from the principal and fellow teachers has made them believe in the importance of encouragement, and in passing it on to others, as expressed by Anaal, an intern, in her response to the questionnaire:

The principal's support made me feel respect and fondness toward my students. I listen to them and talk to them, because that's what I think is right. That's what I learned from the principal's attitude.

#### Expressions of Mental Burnout and An Impaired Sense of Well-Being

Nine participants (12%) commented that an overload of demands and a lack of support cause a sense of fatigue. This situation has led to negative emotions like guilt and despair, a reduced sense of relatedness and competence, stress, and various forms of burnout.

**Sabil** (an intern, in his interview): You come to school in the morning with an agenda, but [it] only lasts for a few minutes, and then everything gets mixed up. A lot of overload and pressure—teaching, dealing with students, solving discipline issues, talking to parents—it's really exhausting. It never ends. There's no end of the day or beginning. Everything becomes like a circle; I'm always waiting for the weekend.

It is evident that overload and pressure produce a sense of frustration and despair in beginning teachers/interns, and lead them to wonder whether they are suitable for the profession. Musa, an intern, decided to make a career change. He stated in his interview:

It's hard for me as a teacher to endure this overload. It's like a flood, and there's no way for you to defend yourself. I come to school with a lot of stress. I wait for the end of the week, and on Saturday evening, I already start feeling depressed. I'm considering not returning to teaching next year.

### Theme 5: The Bright Side of the Story—Needs Support From the Principal and Fellow Teachers

Alongside the theme of needs suppression, an opposite picture emerges of needs support: A warm, positive attitude; creating a family climate (relatedness support); guidance; available assistance; aiding beginning teachers/interns' strengths; believing in their abilities; positive evaluations (competence support); a respectful attitude; attentiveness; and encouraging choice, creativity, and initiative (autonomy support). In turn, this fosters a sense of needs satisfaction, reinforces one's self-image, and optimal absorption at school. Samira, a beginning teacher, expressed an instance of needs support in her interview:

From the very beginning, I felt [like we were] family. The principal was very supportive and listened to my requests and constraints, and was very patient [with] me. Today, it's easy for me to approach him because he made me feel good.

Some participants mentioned experiences of competence that helped them to face new challenges and to develop a desire to contribute to their school, as expressed by Najoud (a beginning teacher, during her interview) and Suha (a beginning teacher, in her response to the questionnaire):

**Najoud:** The principal welcomed me and showed me the lesson schedule, and told me he'd given me the best classes because he believes in me. I was very happy with the principal's faith and confidence in me, and decided to live up to his expectations.

**Suha:** The staff helps all the time and believes in the professionalism of every teacher. They said I can do what I want, and introduce methods and materials I consider suitable. They told me I have my own special place in the staffroom. I use their help a lot.

Some participants referred to autonomy support from the principal and fellow teachers, which led to greater involvement in school, as described by Lubna (an intern):

The principal is open-minded. He listens a lot. He told me he respects my creativity and that I've got the space to do the things I believe in. That's also the message I get from the staff, so I initiate a lot and constantly introduce changes and new things.

### In Sum: What Did the Participants in the Study Tell Us?

This study mainly presents experiences of needs frustration among Bedouin beginning teachers/interns, along with positive experiences as a kind of mirror image.

The participants described how the principals, veteran teachers, and students treat them. The foremost theme is autonomy suppression. They commented on experiences of exploitation, coercion, a lack of freedom to act, and gender- and culture-based discrimination. They also reported competence suppression. They receive negative messages regarding their teaching ability, negative feedback, a lack of assistance, and face various difficulties in the classroom. Additionally, they do not feel they belong in the staff room. At the same time, when beginning teachers/interns receive needs support, they develop a sense of needs satisfaction, which in turn leads to positive outcomes.

In most cases, the participants drew a clear connection between the suppressive or supportive behaviors of diverse figures at school and their own reactions (e.g., their motivation to invest in their work). Figure 1 maps the results in a category tree. The connections expressed in the diagram are based on the findings and reflect the basic premises of SDT, as well as the results of studies that attest to links between environmental features and motivational, emotional, behavioral, and other outcomes (Ryan and Deci, 2017).

### Discussion

The present study examined the emotional-motivational experiences of Bedouin beginning teachers. Most of their experiences involve needs suppression by authority figures and fellow teachers. However, some of the teachers described their school as a needs-supportive environment. The findings indicate that the majority of beginning teachers/interns (most of whom are women) comprise a disadvantaged, low status group.

These findings align with those of other studies that indicate that experiences of needs suppression are associated with a range of negative outcomes among teachers, while autonomy support promotes autonomous motivation and optimal functioning (Vansteenkiste and Ryan, 2013; Ryan and Deci, 2017). Most of the studies on teacher motivation focus on in-service teachers. Bedouin beginning teachers/interns have now joined this population.

The results highlight the unique characteristics of the induction process in Bedouin schools, the importance of supporting teachers' needs, and the figures affecting their integration into the school. All of these issues are discussed from the angle of SDT, with an emphasis on the unique cultural characteristics of Bedouin society and schools, which are reflected in the teachers' relationships and experiences.

### Induction in Bedouin Schools

The complexity of the induction process, and the difficulties facing beginning teachers around the world, are well-known (Flores, 2017). Research has shown that at the beginning of their career, new teachers experience frustration and discomfort (Pillen et al., 2013). The new teacher faces pedagogic, emotional and social hurdles, and experiences difficult adjustment to the school's organizational culture (Abu Ras, 2010; De Neve and Devos, 2017). The transition from professional training into actual educational work raises questions and doubts revolving around one's personal and professional identity (Pillen et al., 2013).

The new teacher's challenges are multi-dimensional; they are personal as well as professional and ecological (Abu Ras, 2010). The personal dimension is linked to self-concepts (such as a sense of professional competence) and emotional coping in the face of the complex reality. On the professional level, the new teacher must acquire new pedagogic knowledge, learn teaching methods, solve disciplinary and motivational problems and more. The ecological dimension refers to the challenge of facing the school as an organization and dealing with the staff and officeholders. The teacher must familiarize himself with policies and protocols and meet the demands of senior teachers, administrators, students and parents (Pritzker and Chen, 2010).

Bedouin beginning teachers/interns face numerous challenges, much like their colleagues from other societies and cultures. According to the few studies conducted among Arab beginning teachers in Israel (Abu Ras, 2010; Iliyan and Zidan, 2011; Schatz-Oppenheimer, 2012), they face diverse hurdles at the professional-pedagogical and organizational levels.

The findings of the present study also point to hindrances typically experienced by beginning teachers. However, Bedouin beginning teachers/interns also have to contend with unique problems associated with their traditional-collectivist culture and the complex reality of the Bedouin-Arab education system (Watad Khoury, 2013; Masri-Herzallah and Arar, 2019), all of which gained an outlet of expression in this study. Its uniqueness lies in observing the stumbling blocks of beginning teachers/interns from a cultural perspective through the lens of SDT concepts.

Regarding the composition of their population, Bedouin schools are characterized by a demographic concentration of students and teachers in accordance with their tribal/family affiliation, which impacts the school's relationships and organizational culture (Masri-Herzallah and Arar, 2019). There are tensions in schools where the students and staff come from different families, which manifest in beginning teachers/interns' sense of relatedness. For instance, beginning teachers/interns working in schools where the principal and most of the teaching staff belong to a particular tribe/family (unlike their own) experience discrimination and mistrust. Beginning teachers/interns find it hard to integrate into the teaching staff if it is composed of groups from different families. Beginning teachers/interns whose families are not represented in the staff room experience the most difficulty in terms of this issue.

Another aspect is the organizational culture and centralized educational climate that typify schools in the Bedouin sector, which reflect the patriarchal social structure and dominant status of authority figures (Abu Asbah, 2006; Iliyan, 2013; Watad Khoury, 2013). This is exhibited in relationships with the students; the study shows that this also applies to relationships with beginning teachers/interns. The findings indicate a relationship style represented by control, an absence of choice, exploitation by authority figures, and additional aspects of autonomy suppression. This relationship style is replicated in beginning teachers' relationships with the students.

Gender-based autonomy suppression, which epitomizes Bedouin culture wherein the status of women is inferior to that of men, is yet another facet associated with Bedouin society's unique character. This cultural quality seeps into the schools. Female beginning teachers experience discrimination associated with them being young women. Some participants defined themselves as a triple minority: they are women, beginning teachers (a minority at the school), and working in an unfamiliar tribal/family environment to which they do not belong. In the study, a new minority group was identified: Bedouin beginning teachers/interns who perceive themselves as a minority within their own cultural community, separately from the minority experience generally reported in the political-national context.

Suppression of the need for competence is displayed in the principals' demand for beginning teachers to teach in accordance with the frontal teaching method accepted at Bedouin schools, which reflects Bedouin society's collectivist nature, and is characterized by the transfer of knowledge, with little choice involved (Iliyan, 2013). Teachers who do not comply are criticized, and the attitude toward them constitutes suppression of their need for competence.

### The Importance of Needs Support and the Need for Autonomy Among Bedouin Teachers

The findings clearly show that needs frustration (especially the need for autonomy) has serious consequences for beginning teachers/interns' functioning at school. Conversely, when their psychological needs are supported, they experience needs satisfaction that increases their desire to contribute to and invest in their schools.

These outcomes run counter to the arguments of researchers that hold a cultural relativism approach. They challenge the universality of SDT, claiming that the need for autonomy is a Western ideal that is not important in traditional Eastern cultures. According to these claims, in collectivist societies, autonomy support will not have the positive effects, and autonomy suppression will not have the negative effects, found in Western societies (e.g., Iyengar and DeVoe, 2003; Liu and Flick, 2019).

The results of the present study underscore SDT's universality claim (Ryan and Deci, 2017) and are consistent with the findings of SDT-based studies carried out in countries with collectivist Eastern societies, some of which made comparisons to Western societies, such as South Korea (Jang et al., 2009), Turkey (Chirkov et al., 2003), China, Hong Kong and Japan (Nalipay et al., 2020). Some studies have even demonstrated the applicability of SDT in the Bedouin society (e.g., Kaplan et al., 2014; Kaplan and Madjar, 2017; Kaplan, 2018).

However, most investigations that examine cultural issues (including in Bedouin society) have primarily focused on school and university students, not beginning teachers. The significance of this study lies in its focus on the unique group of Bedouin teachers working in schools whose population composition, and the character of the relationships within them, reflect a collectivist-patriarchal society.

### Meaningful Figures in the Lives of Beginning Teachers/Interns

The study implies that one of the greatest challenges facing beginning teachers/interns is not inside the classroom, but outside it; namely, their relationships with school staff, especially the principal. In the study, the principal is portrayed as a central figure that influences beginning teachers/interns' psychological experiences. Needs suppression by the principal produces negative emotional experiences and reduces the intensity and quality of beginning teachers/interns' motivation. The present study joins previous ones (e.g., Eyal and Roth, 2011) that point to a leadership style typified by autonomy suppression, which impairs autonomous motivation and well-being and increases one's sense of burnout. In contrast, autonomy support from principals forecasts a sense of needs satisfaction, which in turn predicts positive outcomes (Klassen et al., 2012).

The importance of the principal in improving the absorption of beginning teachers has been shown in recent studies carried out in Israel, which highlight the merit of supporting the autonomy of teachers in general, and of beginning teachers in particular (from non-SDT theoretical angles), such as involving teachers in decision-making and maintaining an open emotional dialogue (e.g., Masri-Herzallah and Arar, 2019). The management style in Bedouin society reflects its collectivist-patriarchal nature, which is authoritative, avoids emotional dialogue, lacks collaboration and is characterized by control (Masri-Herzallah and Arar, 2019). These studies reinforce the results of the present study, which mainly stresses aspects of psychological needs suppression by principals, alongside experiences of needs support and its positive effects.

Other crucial figures in the lives of beginning teachers/interns are veteran teachers. The study revealed that they play a critical role in supporting or suppressing beginning teachers/interns' needs. Examination of veteran teachers' influence on beginning teachers/interns' sense of needs satisfaction is not a central facet in the literature. However, a few studies refer to it as one of the stress factors for beginning teachers, such as Pelletier et al. (2002), who revealed that one of the stress factors for beginning teachers is the need to adapt to the teaching methods of veteran teachers.

### The Implications of Beginning Teachers/Interns' Needs Frustration

The results suggest that needs suppression affects diverse outcomes for beginning teachers/interns, one of which is motivation in terms of intensity and quality. Thus, an experience of needs frustration leads to controlled motivation. These findings join previous ones that imply an experience of needs frustration predicts a range of negative ramifications among teachers (Vansteenkiste and Ryan, 2013). For instance, school pressures (e.g., curriculum-related demands, pressure from principals, or negative student behaviors) frustrate teachers' needs and adversely impact autonomous motivation (Pelletier et al., 2002). Another component of impaired motivation emerged from the participants' descriptions of a sense of burnout and reduced well-being. They reported numerous negative sentiments such as anger, anxiety, shame, guilt, a sense of coercion, and a sense of burnout.

Previous studies underscore the link between teachers' experience of needs satisfaction and autonomous motivation, and positive emotional experiences (e.g., Abos et al., 2018). Needs satisfaction and autonomous motivation are positively correlated with a sense of self-actualization, and negatively tied to emotional burnout (Kaplan and Madjar, 2017).

Another consequence of needs support or frustration is the effect of teachers' experience on attitudes toward others; in this case, students. The study revealed that when beginning teachers/interns experience suppression, they transfer it to their students. Preceding research has also encountered such outcomes. For example, Abos et al. (2018) showed that when teachers feel pressured, they are more likely to pressure their students. In another study, teachers' controlled motivation predicted their autonomy-suppressive behaviors (Soenens et al., 2012). Pelletier et al. (2002) described how pressure from above (from principals, pressure to meet external standards, etc.) led to teachers being more controlling in their demeanor toward their students.

### The Contribution of the Study to SDT and Its Applicability in Education

Unlike qualitative-positivist research, which is the basis for most SDT studies (Han and Yin, 2016; Chirkov and Anderson, 2018), a qualitative phenomenological research allows the researcher to closely examine the meaning people give to their lives (Creswell and Poth, 2017). The current study focuses on the experiences of beginning teachers in a particular context: schools in the Bedouin society. Closeness to the lives of participants allowed the researcher to elicit authentic personal experiences, thus making SDT a living theory. The study provides descriptions of the typical working environment of teachers and introduces personal stories that demonstrate experiences of need frustration or need satisfaction, as well as the effect of the environment on the teachers' motivation, investment, well-being and more. These findings bring SDT principles to life, showing not only that supporting needs is crucial for the optimal functioning of people—beginning teachers in this case—but also providing evidence for the motivational mechanisms at the basis of the theory (see Figure 1).

The qualitative methodology bridges between SDT and the actual internal and external reality of the research participants. Thus, the attempts to implement theoretical insights in educational practice may benefit from the study (Kaplan et al., 2012). Specifically, the findings may encourage an implementation of SDT in the cultural context of the Bedouin society in particular, and in collectivistic societies at large. Each cultural context is unique; consequently, as maintained in the qualitative paradigm, reality is multifaceted (Shkedi, 2003). Thus, having established the core principles of SDT (Ryan and Deci, 2017), it is now time to thoroughly study specific cultural contexts in order to promote the theory's implementation.

According to SDT, psychological needs are universal and therefore will be expressed in all cultures, which indeed has been demonstrated in multiple studies (Ryan and Deci, 2017). Yet the theory maintains that specific experiences of support or frustration of needs and expressions of need satisfaction may differ across cultures (Deci and Ryan, 2000). The current study strengthens the universality claim of the theory, while also demonstrating unique human experiences, which can only be understood through qualitative research. The methodology employed in this study can be used to understand the unique characteristics of specific contexts, which is crucial for implementing SDT. Therefore, in addition to applying general, research-derived principles (such as encouraging choice and promoting relevancy in order to support autonomy; or providing non-judgmental feedback in order to promote competence), it is important to address the community's unique cultural characteristics. For example, in light of the findings of the current study, supporting teachers' sense of relatedness requires examining the composition of the school faculty and encouraging better acquaintance of teachers of different tribes and families. Furthermore, when planning intervention, it is important to understand the patriarchal-hierarchical structure that underlies the relationships between the principal or other authority figures and beginning teachers in the particular culture.

In a society where teacher motivation is of concern to educators, SDT provides a theoretical and practical framework that may lead to positive changes. The study presents tools that may be used by scholars and educators attempting to study different cultures or plan SDT-based interventions. There is a small number of studies that have focused on beginning teachers' motivation from an SDT perspective, and even fewer have done so through qualitative methods (Fernet et al., 2016; Kaplan et al., 2016). The current study contributes to that knowledge, as it focuses on the Bedouin culture and the population of beginning teachers, which have been scarcely studied (Kaplan and Madjar, 2017).

### Practical Recommendations

The environment and context in which individuals act have a fundamental impact on the nature of their emotional-motivational processes (Deci and Ryan, 2000). Consequently, any intervention program must take into account the behaviors that support each psychological need (Reeve, 2006; Kaplan and Madjar, 2017). Supporting beginning teachers/interns' autonomy can include encouraging choice, advancing initiatives based on teachers' fields of interest, stressing beginning teachers/interns' success, and, of course, refraining from suppressive processes, as shown in the present study. Relatedness support can include familiarization with school staff, creating relationships based on trust, and frameworks to facilitate support. Competence support can include non-judgmental feedback, setting optimal challenges, and providing help. These are just a few examples.

In light of the difficulties faced by beginning teachers, the Israel Ministry of Education has built a support framework for beginning teachers, which includes a specialized workshops (that extends over 2 years) and mentoring by an experienced teacher. My recommendation is to integrate SDT principles into similar workshops, especially those attended by Bedouin teachers. In addition, it is important to train mentors to provide need-supportive mentoring.

The study shows that system-wide intervention is needed in the schools. To build a needs-supportive school setting, it is critical to promote an organizational outlook on processes that advance optimal functioning and autonomous motivation among beginning teachers/interns. In schools, it is vital to have an open dialogue about the psychological needs of beginning and veteran teachers, and to construct an environment that responds to those needs.

As part of a school-wide effort, it is important to enhance the knowledge of teachers and other school personnel in SDT-related issues addressed by the current study. These may include the following issues: How may self-determined teaching lead to self-determined learning (Roth et al., 2007)? How might a sense of coercion among teachers affect their teaching motivation or yield a controlling teaching style? Why do teachers adopt a controlling teaching style toward students and how can they become more autonomy supportive (Reeve, 2009)? And finally, how does a principal's leadership style affect teachers' motivation (Eyal and Roth, 2011)?

Working with principals is indispensable. It is crucial for principals to become familiar with principals for creating an optimal environment for absorbing beginning teachers/interns. In the principals' training process, it is necessary to address a transition from traditional hierarchical leadership to autonomy-supportive leadership (Eyal and Roth, 2011).

Our findings reflect need-frustration among beginning female teachers. It is important to keep in mind that the Bedouin society is undergoing changes (Sharabi, 2014). When women set out to enter the workforce and become teachers, they are paving a path for themselves through which they can overcome the discriminatory traditional life course and advance toward social mobility (Seginer and Mahajna, 2004). The findings indicate that even when women break through, they continue to experience suppression. It is therefore important to support them through this change. Workshops that address their psychological needs can help them build resilience and provide important resources (Vansteenkiste and Ryan, 2013). This recommendation is aimed toward the Ministry of Education but also toward other community frameworks that support women's empowerment.

One of the issues troubling educators with regard to Bedouin schools is the matter of motivation. The Bedouin education system faces an array of obstacles, most of which are cultural, that affect teachers' motivation (Masri-Herzallah and Arar, 2019). This study indicated these difficulties from the perspective of BPNT, and demonstrated how the behaviors of different figures at school can affect teachers' motivation. This has led me to suggest that principals engage in emotional-motivational processes, and develop an environment that enhances teachers' autonomous motivation. A needs-supportive environment can reduce the dropout rate of beginning teachers—a familiar phenomenon in many countries around the world (Sperling, 2015)—and that can cultivate quality teachers who support their students' autonomy (Roth et al., 2007).

### Limitations of the Study and Recommendations for Future Research

The findings of the present study are based on a sample from one college of education. Future research should expand the sample to include additional colleges of education in order to prevent possible context bias. It would also be interesting to include a sample of teachers belonging to an individualistic culture, and to compare the experiences of teachers from various societies.

Another limitation concerns the cultural characteristics of the Bedouin society. We based this study on theoretical and empirical literature that defines the Bedouin culture as collectivist-hierarchical and paternalistic (e.g., Nasser-Abu Alhija and Israelashvili, 2021). In light of the changes that the Bedouin society has been undergoing, it would be interesting to use an accepted measure to examine its cultural characteristic from the perspective of the educated young adults that participated in the study (see, for example, Chirkov et al., 2003).

Future quantitative studies might explore the characteristics of the management style of principals in Bedouin society, and how this style affects the absorption of beginning teachers. Another interesting approach would be to examine the issues from the students' perspectives (e.g., how they experience the integration of beginning teachers into the school).

### Some Words of Conclusion

The research has emerged because of the need to give a platform to the silenced voices of beginning teachers/interns. The study enables a better understanding of teachers' reality and their inner world. It does not intend to criticize school principals or staff. Its aim is to promote change in the complex reality of the schools by creating a needs-supportive environment.

## Data Availability Statement

The raw data supporting the conclusions of this article will be made available by the author, without undue reservation.

## Ethics Statement

The studies involving human participants were reviewed and approved by Kaye Research Autoroty. The patients/participants provided their written informed consent to participate in this study.

## Author Contributions

The author confirms being the sole contributor of this work and has approved it for publication.

## Conflict of Interest

The author declares that the research was conducted in the absence of any commercial or financial relationships that could be construed as a potential conflict of interest.

## Footnotes

^1^The term *suppression* (rather than *thwarting*) is used to reflect the reality described in this study, in which beginning teachers are controlled by a strong, dominant figures, whose behavior undermines their experience of need satisfaction.
